# SNP Typing for Germplasm Identification of *Amomum villosum* Lour. Based on DNA Barcoding Markers

**DOI:** 10.1371/journal.pone.0114940

**Published:** 2014-12-22

**Authors:** Qionglin Huang, Zhonggang Duan, Jinfen Yang, Xinye Ma, Ruoting Zhan, Hui Xu, Weiwen Chen

**Affiliations:** 1 Research Center of Chinese Medicinal Resource Science and Engineering, Guangzhou University of Chinese Medicine, Guangzhou, Guangdong Province, China; 2 Key Laboratory of Chinese Medicinal Resource from Lingnan, Ministry of Education, Guangzhou, Guangdong Province, China; 3 Life Science Department, Huizhou University, Huizhou, Guangdong Province, China; University of Hong Kong, China

## Abstract

*Amomum villosum* Lour., produced from Yangchun, Guangdong Province, China, is a *Daodi* medicinal material of Amomi Fructus in traditional Chinese medicine. This herb germplasm should be accurately identified and collected to ensure its quality and safety in medication. In the present study, single nucleotide polymorphism typing method was evaluated on the basis of DNA barcoding markers to identify the germplasm of Amomi Fructus. Genomic DNA was extracted from the leaves of 29 landraces representing three *Amomum* species (*A*. *villosum* Lour., *A. xanthioides* Wall. ex Baker and *A. longiligulare* T. L. Wu) by using the CTAB method. Six barcoding markers (ITS, ITS2, *LSU* D1–D3, *matK, rbcL* and *trnH-psbA*) were PCR amplified and sequenced; SNP typing and phylogenetic analysis were performed to differentiate the landraces. Results showed that high-quality bidirectional sequences were acquired for five candidate regions (ITS, ITS2, *LSU* D1–D3, *matK*, and *rbcL*) except *trnH-psbA*. Three ribosomal regions, namely, ITS, ITS2, and *LSU* D1–D3, contained more SNP genotypes (STs) than the plastid genes *rbcL* and *matK*. In the 29 specimens, 19 STs were detected from the combination of four regions (ITS, *LSU* D1–D3, *rbcL*, and *matK*). Phylogenetic analysis results further revealed two clades. Minimum-spanning tree demonstrated the existence of two main groups: group I was consisting of 9 STs (ST1–8 and ST11) of *A. villosum* Lour., and group II was composed of 3 STs (ST16–18) of *A. longiligulare* T.L. Wu. Our results suggested that ITS and *LSU* D1–D3 should be incorporated with the core barcodes *rbcL* and *matK*. The four combined regions could be used as a multiregional DNA barcode to precisely differentiate the Amomi Fructus landraces in different producing areas.

## Introduction

*Amomum villosum* Lour. belonging to the monophyletic Zingiberaceae family is a valuable herbaceous plant in southern China [1]. The ripe fruits of *A. villosum* Lour., *A. xanthioides* Wall. ex Baker and *A. longiligulare* T.L. Wu are used as Amomi Fructus, one of the traditional Chinese medicines. Amomi Fructus is embodied in the Chinese pharmacopeia because this traditional medicine elicits significant effects, such as eliminating dampness, promoting appetite, regulating the flow of *Qi* (in traditional Chinese medicine, *Qi* is the most essential, active, but invisible substance that constitutes the body and maintains life activities), preventing miscarriage, warming the spleen, and curing diarrhea [2]. Amomi Fructus produced from *A. villosum* Lour. in Yangchun City, located in Guangdong Province, is considered a *Daodi* (“genuine”) medicinal material [1], [3] and is thus sold at a higher price. However, ripe fruits of *A. villosum* Lour. produced in Guangxi Province and Yunnan Province, as well as those of *A. xanthioides* Wall. ex Baker or *A. longiligulare* T.L. Wu, are also traded as *Daodi* medicinal materials of Amomi Fructus because of highly similar morphological traits. Therefore, Amomi Fructus landraces should be identified using an accurate and reliable method to ensure quality and safety in medication.

DNA barcoding is a new technology in which a standard DNA region is used to identify species [4]. In 2009, the Plant Working Group of the CBOL recommended the use of two plastid regions, namely, *rbcL* and *matK*, as core barcodes of land plants [4]. Some researchers later proposed the use of ITS (or ITS2, a subset of ITS) and *trnH-psbA* as supplementary barcoding loci to increase the discriminatory power of this method [5]–[9]. The large subunit (LSU) of nuclear ribosomal DNA (rDNA) contains 12 expansion segments (D1–D12) [10], which may provide targets for a suitable barcode; for example, *LSU* D1–D2 has been successfully used to identify yeasts [11] and fish [12] and 26S rDNA D1–D3 (*LSU* D1–D3) has been used to identify medicinal plants [13]. DNA barcoding is a useful molecular tool used to authenticate medicinal plants [6], [9], [13]–[17]. However, the discriminatory ability of these candidate barcodes has not been thoroughly evaluated using *Daodi* medicinal materials and related substituents.

Single nucleotide polymorphism (SNP) is a single nucleotide variation in a particular and defined genetic location in at least 1% of the population [18], [19]. SNP is one of the most abundant, stable genetic polymorphisms in a genome; this polymorphism is applicable to resolve differences among closely related species [20], [21]. With the improvement of polymerase chain reaction (PCR) and DNA sequencing, SNP typing has been successfully used to accurately and conveniently identify medicinal plants [22]–[25], botanical origin [26], and bacteria [27]–[30]. In our study, SNP typing method was evaluated using six candidate DNA barcoding markers (ITS, ITS2, *LSU* D1–D3, *matK*, *rbcL*, and *trnH-psbA*) to identify three original species (*A. villosum* Lour., *A. xanthioides* Wall. ex Baker, and *A. longiligulare* T.L. Wu) of Amomi Fructus. Phylogenetic trees based on the concatenated SNP data of ITS–*LSU* D1–D3–*rbcL*–*matK* were also constructed.

## Materials and Methods

### Collection of plant materials

**Figure 1:**
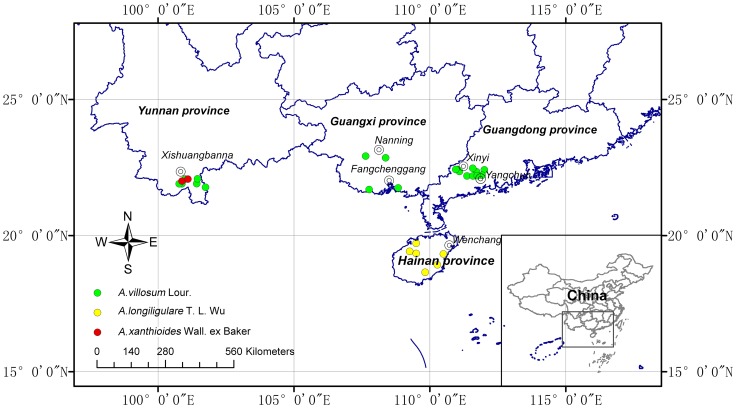
Geographic origins of 29 landraces of Amomi Fructus (*A. villosum* Lour., *A. xanthioides* Wall. ex Baker and *A. longiligulare* T. L. Wu) sampled in this study. The locations of samples are illustrated on the map based on longitudes and latitudes.

Three original plants of Amomi Fructus were sampled from July 2010 to August 2014 (Fig. 1, S1 Table) in China. *A. villosum* Lour. (n = 21) individuals, including seven landraces (AV01 to AV07) of *Daodi* medicinal materials obtained from Yangchun City in Guangdong Province, were collected from 20 culturing areas of Guangdong Province, Guangxi Province, and Yunnan Province. We selected AV03 as an authoritative specimen of *A. villosum* Lour. because this plant originated from Jinhuaken of Panlong Town, Yangchun City, an area where Amomi Fructus is the best, as recorded in the book of *Yao Wu Chu Chan Bian* by Chen-renshan in 1930. *A. xanthioides* Wall. ex Baker (n = 2) individuals were collected from Xishuangbanna City of Yunnan Province. *A. longiligulare* T.L. Wu (n = 6) individuals were gathered from Wenchang City, Danzhou City, Wanning City, and Lingshui City, Hainan Province. No specific permissions were required in sampling because these plants are not considered endangered. Our voucher specimens were verified by Prof. Ruo-Ting Zhan (Guangzhou University of Chinese Medicine, China) and preserved at the Research Center of Chinese Herbal Resource Science and Engineering, Guangzhou University of Chinese Medicine, China.

### DNA extraction, PCR amplification, and sequencing

Genomic DNA was extracted from gel-dried leaves by using the CTAB method as previously described by Huang *et al*. [31]. Six loci were amplified and sequenced from two individuals of each specimen by using a pair of universal primers (Table 1). PCR amplification was conducted in a 50 µL total reaction volume containing 0.2 mM of dNTPs, 0.25 mM of each primer, 10 mM of Tris-HCl (pH 8.3), 50 mM of KCl, 1.5 mM of MgCl_2_, 3 U of Ex *Taq* DNA polymerase (Takara, Japan), and approximately 75 ng of template DNA. PCR amplification was performed in a Peltier thermal cycler C1000 (BioRad, Hercules, CA, USA) in accordance with the reaction protocol described in Table 1. PCR products were evaluated in 1.0% agarose gel prepared in 1× TAE (40 mM of Tris-acetate, 1 mM of Na2EDTA, pH 8.3) electrophoresis buffer and purified using a multifunction DNA purification kit (Bioteke, China). The purified products containing 500 bp to 800 bp DNA segments were bidirectionally sequenced (PCR thermocycling conditions: 96°C for 15 s, 50°C for 15 s, 60°C for 4 min, 25 cycles) using a BigDye Terminator v3.1 Cycle sequencing kit (Applied Biosystems, Foster City, CA, USA) and read using an ABI 3730XL analyzer (Applied Biosystems).

**Table 1:** Primers and PCR reaction conditions used in this study.

Region	Primer name	Sequence	Reaction condition	References
**ITS**	ITS5 (F) ITS4 (R)	5′-GGAAGTAAAAGTCGTAACAAGG-3′ 5′-TCCTCCGCTTATTGATATGC-3′	94°C 5 min; 94°C 30 s, 55°C 30 s, 72°C 1 min, 35 cycles; 72°C 5 min	[36]
**ITS2**	S2F S3R	5′-ATGCGATACTTGGTGTGAAT-3′ 5′-GACGCTTCTCCAGACTACAAT-3′	94°C 2 min; 94°C 30 s, 52°C 30 s, 72°C 1 min, 32 cycles; 72°C 5 min	[6]
***LSU*** ** D1–D3**	DUAN5 DUNA6	5′-TAGTAACGGCGAGCGAAC-3′ 5′-GGCATAGTTCACCATCTTTC-3′	94°C 2 min; 94°C 30 s, 58°C 30 s, 72°C 50 s, 35 cycles; 72°C 7 min	[13]
***trnH-psbA***	trnH2 psbAF	5′-CGCGCATGGTGGATTCACAATCC-3′ 5′-GTTATGCATGAACGTAATGCTC-3′	94°C 2 min; 94°C 30 s, 56°C 30 s, 72°C 50 s, 33 cycles; 72°C 5 min	[37], [38]
***rbcL***	rbcLa-F rbcLa-R	5′-ATGTCACCACAAACAGAGACTAAAGC-3′ 5′-GTAAAATCAAGTCCACCRCG-3′	94°C 3 min; 94°C 30 s, 56°C 30 s, 72°C 50 s, 32 cycles; 72°C 7 min	[4]
***matK***	KF KR	5′-CTCACAATTTACAATCTAGTCATTC-3′ 5′-GAGGATCCACTATGATAGTGAGAA-3′	94°C 2 min; 94°C 30 s, 58°C 30 s, 72°C 50 s, 35 cycles; 72°C 7 min	[31]

### Sequence quality and recoverability

Sequence quality was assessed using Sequence Scanner version 1.0 software (Applied Biosystems) with two quality metrics, trace score (TS) and contiguous read length (CRL). TS, calculated as the average basecall quality value of bases in the post-trim sequences, ranged from 0 to 100 and was defined using three levels: low quality (TS, 0–20), medium quality (TS, 21–34), and high quality (TS, 35–100). CRL is the longest uninterrupted stretch of bases with a quality higher than 20 QV (represents an error rate of basecall at 1 in 100, and a call accuracy of 99%) in a window size of 20 bp. Sequence success rate was examined on the basis of the ratio of sequences traces with TS ≥35 and CRL ≥200 bp to the total number of PCR products.

Sequence traces were trimmed, assembled, and manually edited using Sequencher 5.0 software (Gene Codes, USA) to obtain high-quality bidirectional sequences. Five quality control criteria were sequentially implemented: 1) the sequence trace should have a CRL ≥200 bp and a TS ≥35; 2) heterozygous sites were indicated by the second peak >40% of the first peak; 3) both 5′ and 3′ ends of the sequence were trimmed until less than three bases with quality scores <25 (or ambiguities) in a 25-base window; 4) assembled contigs should have a minimum overlap of 80% in the alignment of forward and reverse reads with a minimum match percentage of 98%; and 5) all of the heterozygous sites (mixed bases) were manually checked and edited on the basis of bidirectional reading chromatograms. Sequence files were deposited in GenBank with the accession numbers KJ151798–KJ151918 and KM411360–KM411384 (S2 Table).

### SNP analysis and typing

SNP sites for each region were identified using Sequencher 5.0 software in accordance with the manufacturer's instructions, and the sequences of the AV03 sample were used as references. Consensus contigs of samples were assembled, trimmed and compared with the reference sequences. The results of variable sites between the sample sequences and the reference sequence were identified as SNPs; and the SNPs of each region in each sample were considered as an SNP genotype (ST). We also concatenated the SNPs of two to four regions to compare the number of SNPs and STs revealed by different multi-region combinations by using MEGA 5 [32].

### Phylogenetic analysis

The concatenated SNPs of ITS–*LSU* D1–D3–*rbcL*–*matK* of 29 landraces were imported in BioNumerics version 7.1 (Applied Maths, Saint-Martens-Latem, Belgium), and an ST number was assigned to each distinct combination of SNPs. Phylogenetic analysis was performed using three clustering methods. Unweighted pair group method using arithmetic mean (UPGMA) was used to generate a dendrogram based on pairwise similarity. Cophenetic correlation value was calculated to determine the branch quality of the dendrogram. This value represents the correlation between the dendrogram-derived similarities and the matrix similarities, thereby providing an estimation of the reliability of a cluster analysis. For maximum parsimony analysis, character data were selected as input data, and characters were treated as categorical. Network creation algorithm was conducted using an optimized maximum parsimony tree (simulated annealing) method, which is a huristic approach used to search for the highest parsimony by simulated annealing. All taxa with zero inter-taxon distance were identified. Root position in the tree was assigned to the deepest branch measured by maximum branch length. Bootstrap values were conducted with 1000 random addition sequence replicates. Considering that the origin of most of the landraces cultivated today can be traced back to the wide cultivation of *A. villosum* Lour. in China in the 1960s [1], we assumed that ancestors' genotype of current landraces remain present in our wide collection of samples. Thus, we further constructed a minimum-spanning tree (MST) to estimate evolutionary relationships by using number of *n*-locus variants (*n* = 1, weight: 10000; n = 2, weight: 10) as priority rule of the algorithm.

## Results

### Assessment of sequence quality and recoverability

**Figure 2:**
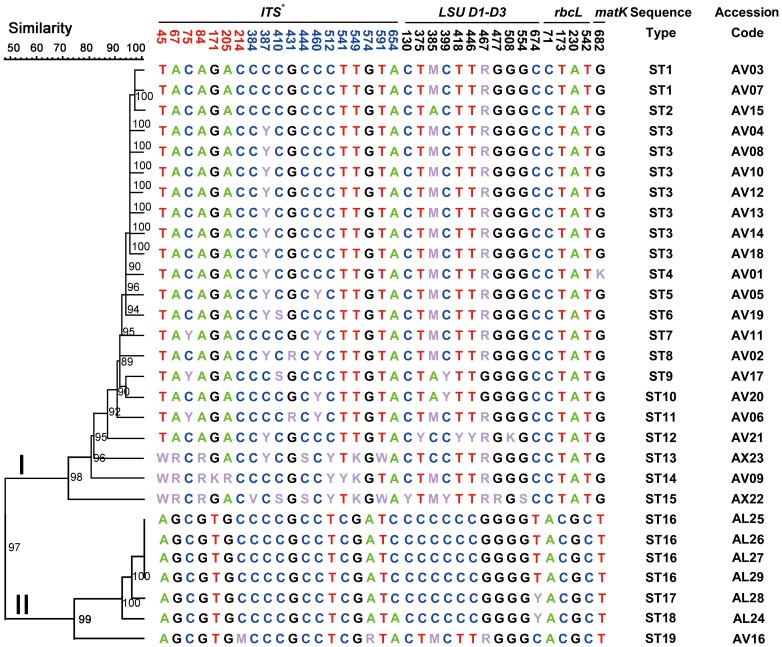
Phylogenetic tree of 29 Amomi Fructus landraces constructed by the UPGMA method based on 35 single nucleotide polymorphisms (SNPs) from four loci (ITS, *LSU* D1–D3, *rbcL*, and *matK*). Numbers on the UPGMA tree branches are bootstrap values (1,000 replicates). Numbers above the bases indicate the position of SNPs in each locus. *The SNPs of ITS included ITS1 (red numbers) and ITS2 (blue numbers). Heterozygous sites were defined according to IUPAC, *i.e.*, W =  A/T; M =  A/C; R =  A/G; Y =  C/T; S =  G/C.

PCR was successfully completed in all of the barcodes; *LSU* D1–D3 exhibited the highest sequencing efficiency of 96.6%, whereas *trnH-psbA* exhibited the lowest sequencing efficiency of 34.5% (Table 2). High-quality bidirectional sequences with a mean TS of ≥51 were obtained from *LSU* D1–D3, ITS, ITS2, *rbcL*, and *matK* in all of the samples. However, only 40 eligible sequences with an average TS of 44 were obtained from *trnH-psbA* possibly because frequent mononucleotide repeats interrupted sequencing reads. The lengths of the acquired sequences ranged from 444 bp for ITS2 to 818 bp for *matK*. Heterozygous base sites (or ambiguous bases, in which exactly two alleles were observed; Fig. 2) were detected in *LSU* D1–D3, ITS (including ITS1 and ITS2), and *matK*, indicating paralogous copies within individuals. All of the heterozygous sites were manually edited and confirmed by bidirectional sequence chromatograms in repeated amplifications and multiple samples.

**Table 2:** Sequencing success, sequence quality, and barcode recovery of six regions.

Region	*LSU* D1–D3	ITS	ITS2	*matK*	*rbcL*	*trnH-psbA*
**No. of samples**	29	29	29	29	29	29
**% PCR success**	100	100	100	100	100	100
**% sequencing success**	96.6 (112/116)	75.9 (88/116)	69.8 (81/116)	78.5 (91/116)	95.7 (111/116)	34.5 (40/116)
**% barcode recovery**	100	100	100	100	100	3.5
**Mean (range) trace score**	51 (42–56)	56 (47–59)	55 (46–59)	52 (47–54)	57 (54–59)	44 (34–53)
**Sequence length (bp) based on trimmed alignments**	714	660	444	818	568	715

### Assessment of discriminatory power

Next, the discriminatory power of the 6 single regions and 9 different combinations of these regions was assessed by analyzing the number of SNPs and STs (Table 3). The values of STs were highly correlated with the number of SNPs (r = 0.9182, *p<*0.01). Discriminatory efficiency varied with different loci and multi-locus combinations. The highest numbers of SNPs and STs among single regions were observed in ITS (19 SNPs and 15 STs), followed by ITS2 (12 SNPs and 14 STs), and *LSU* D1–D3 (11 SNPs and 8 STs). The lowest numbers of SNPs and STs were observed in *trnH-psbA*, with (0 SNPs and only 1 ST, in which only 1 qualifying sequence for *trnH-psbA* was obtained). Furthermore, only a few SNPs and SNP genotypes were observed in the two remaining regions, namely, *rbcL* and *matK* (1 to 4 SNPs and 2 to 3 STs). In combined multiple loci, the highest numbers of SNPs and STs were obtained from ITS + *LSU* D1–D3 + *rbcL*+ *matK* (35 SNPs and 19 STs), followed by ITS + *LSU* D1–D3 (30 SNPs and 18 STs), ITS2 + *LSU* D1–D3 (23 SNPs and 18 STs), and ITS + *rbcL* + *matK* (24 SNPs and 16 STs). By contrast, the lowest numbers of SNPs and STs were detected in *rbcL* + *matK* (5 SNPs and 3 STs).

**Table 3:** Discriminatory power of six single regions and nine multi-locus region combinations in three *Amomum* species from 29 producing areas in China.

Number of regions	Locus or combination	Total number of single nucleotide polymorphisms (SNPs)	Number of SNP genotypes(STs)*
1	ITS	19	15
1	ITS2	12	14
1	*LSU* D1–D3	11	8
1	ITS1	7	6
1	*rbcL*	4	2
1	*matK*	1	3
2	ITS + *LSU* D1–D3	30	18
2	ITS2 +*LSU* D1–D3	23	18
2	ITS1 +*LSU* D1–D3	18	12
2	*rbcL* + *matK*	5	3
3	ITS + *rbcL* + *matK*	24	16
3	ITS2 + *rbcL* + *matK*	17	15
3	*LSU* D1–D3 + *rbcL* + *matK*	16	9
3	ITS1 + *rbcL* + *matK*	12	7
4	ITS + *LSU* D1–D3 + *rbcL* + *matK*	35	19

*Pearson correlation coefficient between the numbers of SNPs and SNP genotypes was 0.9182; the 2-tailed probability value was 0.0000005.

Among 35 SNPs from the four combined loci, 9 sites at 45, 67, 84, 171, 205, 512, 541, 549, and 574 bp in the ITS sequence (KJ151873), 4 sites at 375, 418, 446, and 674 bp in the *LSU* D1–D3 sequence (KJ151800), 4 sites at 71, 173, 230, and 542 bp in the *rbcL* sequence (KJ151848), and 1 site at 682 bp in the *matK* sequence (KJ151824) discriminated *A. longiligulare* T.L. Wu from *A. villosum* Lour. and *A. xanthioides* Wall. ex Baker. Additionally, 5 sites at 45, 67, 84, 512, and 549 bp in the ITS sequence identified all 3 *Amomum* species.

### Phylogenetic analysis

**Figure 3:**
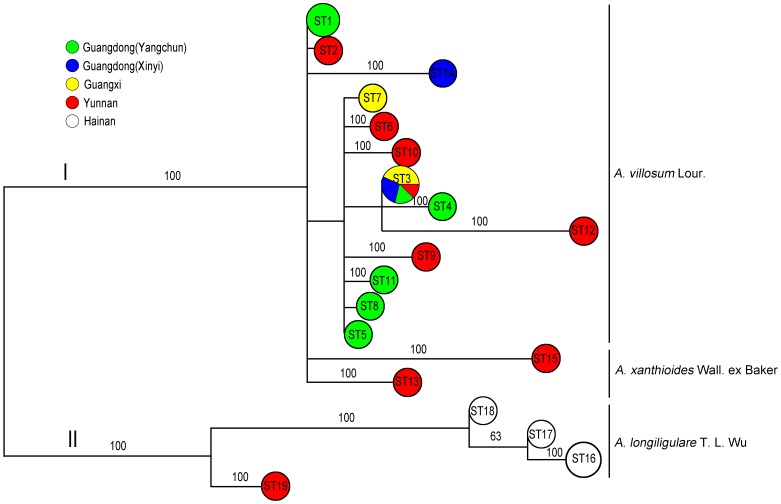
Phylogenetic tree of 29 Amomi Fructus landraces constructed by the maximum parsimony method based on 35 single nucleotide polymorphisms (SNPs) from 4 loci (ITS, *LSU* D1–D3, *rbcL*, and *matK*). Each circle in the tree represents a unique SNP genotype (ST). Accession codes for each ST are presented in Fig. 2. Numbers on the tree branches are bootstrap values from 1,000 replicates. Only bootstrap values >60% are included.

In the UPGMA phylogenetic tree constructed on the basis of 35 concatenated SNPs of ITS–*LSU* D1–D3–*rbcL*–*matK*, all of the specimens formed 19 STs and clustered into two major clades (Fig. 2). Clade I included *A. villosum* Lour. and *A. xanthioides* Wall. ex Baker; clade II consisted of *A. longiligulare* T.L. Wu and one accession(AV16,from Jinha of Xishuangbanna City of Yunnan Province) of *A. villosum* Lour. The maximum parsimony tree (Fig. 3) also revealed two clades similar to the UPGMA tree. Consistent with traditional morphological taxonomy, these results demonstrated that the original Amomi Fructus plants included two species, namely, *A. villosum* Lour. and *A. longiligulare* T.L. Wu; *A. xanthioides* Wall. ex Baker was found as a variant (i.e., *A. villosum* Lour. var *xanthioides* (Wall. ex Bak.) T. L. Wu & S. J. Chen) belonging to *A. villosum* Lour.

**Figure 4:**
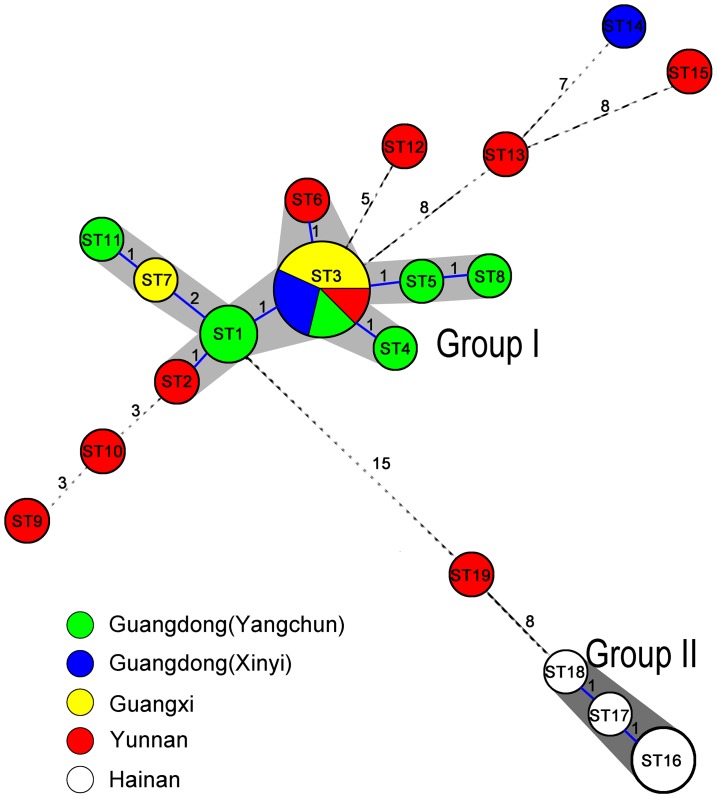
Minimum spanning tree analysis of 29 Amomi Fructus landraces based on the data set of 35 SNPs from four loci (ITS, *LSU* D1–D3, *rbcL*, and *matK*). Each circle in the tree represents a different SNP genotype (ST). The circle size is proportional to the number of landraces belonging to a ST. Numbers between circles represent the number of SNP differences. Two or more STs differing at two or fewer SNPs were regarded as a group (indicated by the gray shadow) and are connected with solid lines; those that differ by more than 2 SNPs are connected with dashed lines.

To visualize genetic relationships among genotypes based on germplasms of Amomi Fructus from different geographic regions, we constructed an MST tree of the 19 STs (ST1–19) and connected these STs based on the numbers of SNP differences. MST tree (Fig. 4) revealed the existence of two main groups (namely group I and II) in which STs differed in two or fewer SNPs with their neighbor node. Group I was the larger, consisting of 9 STs (ST1–8 and ST11) of *A. villosum* Lour., including all of the samples of Guangdong Province(except AV09), Guangxi Province, and three samples of Yunnan Province (AV15, AV18, AV19). Group II was composed of 3 STs (ST16–18) of *A. longiligulare* T.L. Wu from Hainan Province. Seven samples, including six samples of Yunnan Province (AV16, AV17, AV20–23) and one sample of Guangdong Province (AV09), were not included in any group. These results clearly reflected the genetic origin of Amomi Fructus landraces of different geographic regions.

## Discussion

A suitable barcoding project to authenticate the germplasms of Amomi Fructus should provide sufficient polymorphism sites to generate variable sequence types among different landraces, which often originate from the same species or from a closely related species of the same genera. Huang *et al.* [31] reported five variable sites (1 in *matK* and 4 in 26S D1–D3, i.e., *LSU* D1–D3) in the germplasms of Amomi Fructus. In the samples collected from 26 production areas in the current study, 35 SNPs (Fig. 2) were found in four candidate barcodes of ITS (gi 84095191:4655–5354), *LSU* D1–D3 (gi 84095191:5378–6121), *matK* (gi 24634775:1223–2111), and *rbcL* (gi 7525012:54958–55556). Among these SNPs, 30 were novel SNPs (19 in ITS, 7 in *LSU* D1–D3, and 4 in *rbcL*). These findings may allow us to precisely identify the species of Amomi Fructus and trace the origin of samples by using their SNP genotypes. Three plastid regions exhibited poor discriminatory power in Amomi Fructus landraces. Although *trnH-psbA* shows high discriminatory power in many medicinal plants and has been proposed as a candidate DNA barcode for plants [14], [15], high-quality bidirectional sequences were not generated because of sequencing problems [4]. *MatK* and *rbcL* exhibited high sequence quality, but only provided one and four SNPs, respectively, to distinguish between *A. villosum* Lour. and *A. longiligulare* T.L. Wu. Indeed, *matK* and *rbcL* are suitable markers that can be used to identify species [4]; however, these markers can provide few polymorphisms to distinguish individuals within species.

The nuclear ribosomal DNA regions ITS and *LSU* D1–D3 showed more variable sequences than the plastid regions *matK* and *rbcL*. ITS with 19 SNPs found at single locus exhibited the greatest variability; this result confirmed that ITS exhibits high levels of sequence differentiation within species [14] and shows greater discriminatory power than plastid regions [33]. *LSU* D1–D3, the 5′ fragment of 26S rDNA, contains three expansion segments (D1, D2, and D3) from a total of 12 expansion segments (D1–D12) [13]. In our study, *LSU* D1–D3 exhibited 11 SNPs. These results are consistent with those in a previous report demonstrating that expansion segment sequences evolve 1.2 to 3.0 times faster than *rbcL* [10]; indeed, this locus may provide a powerful tool to identify Amomi Fructus germplasms.

Despite the high discriminatory efficiency of ITS and *LSU* D1–D3, single locus may not be used to differentiate >52% of Amomi Fructus varieties; thus, multiple loci are necessary for maximal identification of these varieties. Our findings highlighted the four-locus combination of ITS *+ LSU* D1–D3 *+ matK* + *rbcL*, which provided maximal resolution of varieties with 19 STs (65.5%) among 29 samples.

Seven samples from different production areas, including Guangdong Province (Shuangjiao of Yangchun City, and Baishi and Chitong of Xinyi City), Guangxi Province (Longan and Guangxi Medicinal Plant Garden in Nanning City, and Shangsi in Fangchenggang City), and Yunnan Province (Jinha in Xishuangbanna City), were not successfully distinguished even using a four-locus combination. These samples notably shared an identical SNP genotype (ST3), which possibly resulted from the artificial introduction of this genotype rather than the low discriminatory effect of the four-locus combination. ST3 could be used as an ancestor genotype [34] for the other nodes connected in one to two SNP differences because the MST tree showed that ST3 was located at the center node (Fig. 4). Moreover, this genotype possibly originated in Yangchun City and was widely distributed to other production areas because most of single SNP variants (ST1, 2, 4, and 5) originated from Yangchun in Guangdong Province. This hypothesis is also consistent with a historical record stating that Yangchun is the *Daodi* production area of *A. villosum* Lour. [1].

ITS and *LSU* D1–D3 incorporated in current plant DNA barcoding systems could be used to precisely identify Amomi Fructus germplasms, which comprise samples of closely related species or subspecies originating from different culturing areas. Our results may be used as basis to promote plant DNA barcoding applications. For example, this project might be used to identify medicinal plants often substituted or altered with other morphologically indistinguishable species or varieties [35]. Moreover, species discrimination of *rbcL* + *matK* was only successful in 72% of the cases, and the remaining specimens were identified as the species group (i.e., congeners of closely related species) [4] – our findings may also have implications in the identification of these unresolved species.

## Supporting Information

S1 Table **Geographic origins of 29 landraces of Amomi Fructus (** ***A. villosum*** **Lour.,** ***A. xanthioides*** **Wall. Ex Baker, and** ***A. longiligulare*** **T.L. Wu).**
(DOCX)

S2 Table **GenBank sequence accession numbers.**
(DOCX)
