# Supplementary material for: SNP Typing for Germplasm Identification of Amomum villosum Lour. Based on DNA Barcoding Markers
Source: PLoS One. 2014 Dec 22;9(12):e114940. doi: 10.1371/journal.pone.0114940 (PMC4274006; doi:10.1371/journal.pone.0114940)
Supplement: S1 Table — Geographic origins of 29 landraces of Amomi Fructus ( A. villosum Lour., A. xanthioides Wall. Ex Baker, and A. longiligulare T.L. Wu). (DOCX) [file pone.0114940.s001.docx]

**Table S1. Geographic origins of 29 landraces of Amomi Fructus (*A. villosum* Lour.,** ***A. xanthioides* Wall. ex Baker, and *A. longiligulare* T.L. Wu).**

| **Taxon** | **Voucher number** | **Location(Latitude, longitude)** |
| --- | --- | --- |
| *Amomum villosum* Lour. | AV01 | Jiuzaiken, Yangchun, Guangdong (N22°24.699′, E112°00.259′) |
|  | AV02 | Outong, Yangchun, Guangdong (N22°25.637′, E111°01.153′) |
|  | AV03 | Jinhuaken, Yangchun, Guangdong (N22°11.215′, E111°21.924′) |
|  | AV04 | Shuangjiao, Yangchun, Guangdong (N22°12.667′, E111°55.063′) |
|  | AV05 | Shangling, Yangchun , Guangdong (N22°20.437′, E111°43.002′) |
|  | AV06 | Jilongken, Yangchun, Guangdong (N22°11.271′, E111°35.219′) |
|  | AV07 | *Amomum villosum* Lour. Model Experimental Field in YangChun , Guangdong (N22°11.735′, E111°44.732′) |
|  | AV08 | Baishi , Xinyi , Guangdong (N22°21.156′, E111°05.337′) |
|  | AV09 | Sihe , Xinyi , Guangdong (N22°28.279′, E111°34.628′) |
|  | AV10 | Chitong , Xinyi , Guangdong (N22°25.499′, E110°57.128′) |
|  | AV11 | Fangcheng, Fangchenggang, Guangxi (N21°41.368′, E107°46.057′) |
|  | AV12 | Shangsi, Fangchenggang, Guangxi (N21°14.972′, E108°49.995′) |
|  | AV13 | Longan, Nanning, Guangxi (N22°55.033′, E107°38.220′） |
|  | AV14 | Guangxi Medicinal plant Garden (N22°51.232′, E108°22.394′） |
|  | AV15 | Jinuo, Xishuangbanna, Yunnan (N21°53.942′, E100°52.807′) |
|  | AV16 | Jinha, Xishuangbanna, Yunnan (N22°04.406′, E101°00.041′) |
|  | AV17 | Jinuo, Xishuangbanna, Yunnan ( N21°53.942′, E100°52.807′) |
|  | AV18 | Jinha, Xishuangbanna, Yunnan (N22°04.406′, E101°00.041′) |
|  | AV19 | Yiwu, Xishuangbanna, Yunnan (N21°54.356′, E101°25.577′) |
|  | AV20 | Forestry Administration of Menla, Xishuagbanna, Yunnan (N22°04.740′, E101°27.308′) |
|  | AV21 | Menga, Xishuangbanna, Yunnan (N21°46.307′, E101°53.604′) |
| *Amomum xanthioides* Wall. ex Baker | AX22 | Jinuo, Xishuangbanna, Yunnan (N21°53.942′, E100°52.807′) |
|  | AX 23 | Jinha, Xishuangbanna, Yunnan (N22°04.406′, E101°00.041′) |
| *Amomum longiligulare* T. L. Wu | AL24 | Nanyang, Wenchang, Hainan (N19°19.198′, E110°29.975′) |
|  | AL25 | Nanfeng, Danzhou, Hainan (N19°30.100′, E109°35.153′) |
|  | AL26 | Baoniao new villiage, Danzhou, Hainan(N19°30.735′, E109°30.174′) |
|  | AL27 | Diaoluoshan nature protection area, Lingshui, Hainan (N18°23.200′, E109°53.138′) |
|  | AL28 | Xinglong, Wanning, Hainan (N18°44.067′, E110°11.650′) |
|  | AL29 | Nanfeng, Danzhou, Hainan (N19°30.104′, E109°35.173′) |
