# Supplementary material for: SNP Typing for Germplasm Identification of Amomum villosum Lour. Based on DNA Barcoding Markers
Source: PLoS One. 2014 Dec 22;9(12):e114940. doi: 10.1371/journal.pone.0114940 (PMC4274006; doi:10.1371/journal.pone.0114940)
Supplement: S2 Table — GenBank sequence accession numbers. (DOCX) [file pone.0114940.s002.docx]

**Table S2. GenBank sequence accession numbers.**

| **Taxon** | **Voucher number** | ITS | ITS2 | *LSU* D1-D3 | *matK* | *rbcL* | *trnH-psbA* |
| --- | --- | --- | --- | --- | --- | --- | --- |
| *Amomum villosum* Lour. | AV01 | KJ151871 | KJ151895 | KJ151798 | KJ151822 | KJ151846 |  |
|  | AV02 | KJ151872 | KJ151896 | KJ151799 | KJ151823 | KJ151847 |  |
|  | AV03 | KJ151873 | KJ151897 | KJ151800 | KJ151824 | KJ151848 |  |
|  | AV04 | KJ151874 | KJ151898 | KJ151801 | KJ151825 | KJ151849 |  |
|  | AV05 | KJ151875 | KJ151899 | KJ151802 | KJ151826 | KJ151850 |  |
|  | AV06 | KJ151876 | KJ151900 | KJ151803 | KJ151827 | KJ151851 |  |
|  | AV07 | KJ151877 | KJ151901 | KJ151804 | KJ151828 | KJ151852 |  |
|  | AV08 | KJ151878 | KJ151902 | KJ151805 | KJ151829 | KJ151853 |  |
|  | AV09 | KJ151879 | KJ151903 | KJ151806 | KJ151830 | KJ151854 |  |
|  | AV10 | KJ151880 | KJ151904 | KJ151807 | KJ151831 | KJ151855 |  |
|  | AV11 | KJ151881 | KJ151905 | KJ151808 | KJ151832 | KJ151856 |  |
|  | AV12 | KJ151882 | KJ151906 | KJ151809 | KJ151833 | KJ151857 |  |
|  | AV13 | KJ151883 | KJ151907 | KJ151810 | KJ151834 | KJ151858 |  |
|  | AV14 | KJ151884 | KJ151908 | KJ151811 | KJ151835 | KJ151859 | KJ151870 |
|  | AV15 | KJ151885 | KJ151909 | KJ151812 | KJ151836 | KJ151860 |  |
|  | AV16 | KJ151886 | KJ151910 | KJ151813 | KJ151837 | KJ151861 |  |
|  | AV17 | KJ151887 | KJ151911 | KJ151814 | KJ151838 | KJ151862 |  |
|  | AV18 | KJ151888 | KJ151912 | KJ151815 | KJ151839 | KJ151863 |  |
|  | AV19 | KJ151889 | KJ151913 | KJ151816 | KJ151840 | KJ151864 |  |
|  | AV20 | KJ151890 | KJ151914 | KJ151817 | KJ151841 | KJ151865 |  |
|  | AV21 | KJ151891 | KJ151915 | KJ151818 | KJ151842 | KJ151866 |  |
| *Amomum xanthioides* Wall. ex Baker | AX22 | KJ151892 | KJ151916 | KJ151819 | KJ151843 | KJ151867 |  |
|  | AX23 | KJ151893 | KJ151917 | KJ151820 | KJ151844 | KJ151868 |  |
| *Amomum longiligulare* T. L. Wu | AL24 | KJ151894 | KJ151918 | KJ151821 | KJ151845 | KJ151869 |  |
|  | AL25 | KM411370 | KM411375 | KM411380 | KM411365 | KM411360 |  |
|  | AL26 | KM411371 | KM411376 | KM411381 | KM411366 | KM411361 |  |
|  | AL27 | KM411372 | KM411377 | KM411382 | KM411367 | KM411362 |  |
|  | AL28 | KM411373 | KM411378 | KM411383 | KM411368 | KM411363 |  |
|  | AL29 | KM411374 | KM411379 | KM411384 | KM411369 | KM411364 |  |
